# Cone-beam computed tomographic evaluation of styloid process: a retrospective study of 498 patients with maxillofacial diseases

**DOI:** 10.1186/s12880-024-01516-w

**Published:** 2024-12-06

**Authors:** Zhang Yang, Jing Yuzi, Liang Beibei

**Affiliations:** 1 Department of Radiology, School and Hospital of Stomatology, Hebei Medical University, Shijiazhuang, 050017 PR China; 2 School and Hospital of Stomatology, Hebei Medical University, Shijiazhuang, 050017 PR China

**Keywords:** Elongated styloid process, Cone-beam computed tomography, Eagle’s syndrome, Styloid length, Styloid angulation

## Abstract

**Purpose:**

The purpose of this study was to assess the structural characteristics of the styloid process (SP) using cone-beam computed tomography (CBCT) examination in patients with maxillofacial diseases. The study aimed to determine the prevalence of elongated styloid process (ESP) and its relationship to gender in the study population. Radiographic records of 498 subjects were evaluated retrospectively. Radiological examinations including measurements of the structure, length, volume, and angulations of styloid process were performed on CBCT images. Males had significantly longer styloid process in both sides than females in the study population and a strong positive linear relationship was found between left and right sides’ process length (*r* = 0.83; *p* < 0.001). The mean internal oblique angle of styloid process differed between genders, but there were no statistically significant differences in the mean anterior oblique angle. Out of 498 subjects, 62 (24.13%) females and 101 (41.91%) males had elongated left styloid process (≥ 30 mm), while 75 (29.18%) and 90 (37.34%) had right side respectively.

**Methods:**

Radiographic records of 498 subjects were evaluated retrospectively. Radiological examinations including measurements of the structure, length, volume, and angulations of styloid process were performed on CBCT images.

**Results:**

Males had significantly longer styloid process in both sides than females in the study population and a strong positive linear relationship was found between left and right sides’ process length (*r* = 0.83; *p* < 0.001). The mean internal oblique angle of styloid process differed between genders, but there were no statistically significant differences in the mean anterior oblique angle. Out of 498 subjects, 62 (24.13%) females and 101 (41.91%) males had elongated left styloid process (≥30 mm), while 75 (29.18%) and 90 (37.34%) had right side respectively.

**Conclusions:**

This study presents the CBCT as an alternative method to CT or panoramic radiographs for the measurement and the assessment of the styloid process. Within the study in 498 subjects in China, it was observed that the males, on average, had significantly longer styloid process and narrower internal oblique angle than females either in left or right side. Around 33% of the study population had ESP.

**Supplementary Information:**

The online version contains supplementary material available at 10.1186/s12880-024-01516-w.

## Introduction

The styloid processes (SP) are thin bony projections located at the base of the skull between the internal carotid artery (ICA) and external carotid artery (ECA) [1]. Abnormalities in the styloid process, such as elongation, angulation anomalies, or calcified stylohyoid or stylomandibular ligaments, can lead to a series of clinical symptoms including pain and paresthesia in the neck, jaw, head, throat, ear, teeth, tongue, and globus sensation [2]. Other symptoms may include eye twitching, hoarseness or voice changes, and cranial nerve injury/irritation [3]. Dentists and otolaryngologists diagnose patients with these symptoms as having Eagle's syndrome.

Eagle's syndrome is a collection of symptoms caused by anomalies of the styloid process that provoke irritation of the structures in the carotid space [4]. It has been suggested in some studies that one-third of the population and three out of four individuals with temporomandibular disorder (TMD) may have an elongated styloid process (ESP) [5]. An ESP is defined as a styloid process longer than 30 mm [6].

Cone-beam computed tomography (CBCT) was initially used for vascular imaging but has also been applied to maxillofacial imaging [7]. The use of CBCT for measuring anatomical structures in the craniofacial region has been in practice for over two decades [8]. CBCT has been proven to be a reliable tool in diagnosing oral and craniofacial diseases. It can be easily installed, has a high spatial resolution, and a potentially lower radiation dose when compared with multislice computed tomography or a series of plain x-rays. CBCT offers advantages such as shorter examination time for patients, and lower costs compared to traditional CT, making it a routine imaging modality for oral and maxillofacial procedures [9]. Panoramic radiographic examinations can detect the length of the styloid process, but there are limitations in obtaining data on the angulations and volume of the styloid process [10]. Multidetector computed tomography (MDCT) is mostly used for measuring cross-sectional images of the whole body and soft tissues, but its three-dimensional imaging capability is relatively limited, making it ineffective for accurately measuring the volume of the styloid process [11].

There have been some studies on the relationship between styloid process morphology and age, as well as gender [12]. Many scholars have conducted relevant measurements of the styloid process using spiral CT. Previous literature has indicated that the length of the styloid process is of significant reference value in the diagnosis of related diseases in the head and neck region [13]. Studies have shown [2, 14] that the angle of styloid process deviation is closely related to symptoms. For example, an excessively large anterior or medial inclination angle (> 40°) can irritate the tonsillar fossa and pharyngeal lateral wall, causing a foreign body sensation in the throat. An excessively small anterior inclination angle (< 20°) may compress nerve endings, leading to pharyngeal pain, while an excessively small medial inclination angle (< 20°) can compress or rub against the carotid arteries in the neck, affecting blood circulation and causing ear pain, pain in the corresponding area, and tinnitus. However, many studies have only focused on patients with SPS, these studies not only have small sample sizes but also measure a limited number of parameters, resulting in an excessively wide reference range for normal styloid processes and excessively large differences in measurement results, which are not representative.

Although there are numerous reports on the use of panoramic x-rays or CT for measuring SP length and examining Eagle's syndrome [10, 15], limited research has been done based on CBCT. In this study, CBCT examinations were used to evaluate the length and medial and anterior inclination angles of the SP [16, 17]. The goals of this study are to assess the length, mesial inclination angle, and anterior inclination angle of the styloid process using CBCT scans [18]. Additionally, the study aims to determine the prevalence of elongated styloid process (ESP) and evaluate the relationship between SP incidence and various factors such as gender and place of residence.

### Materials and methods

A total of 498 patients with oral diseases in Hebei Province, China, who underwent CBCT examination at the dentistry department between January 2019 and January 2020, were retrospectively evaluated in this study. The study followed the principles outlined in the Declaration of Helsinki, including all amendments and revisions. The research was approved by the Medical Ethics Committee of Hospital of Stomatology, Hebei Medical University (NO. [2018] 033). Informed written consent was obtained from all participants after an explanation of the nature of the study, as approved by the Medical Ethics Committee of Hospital of Stomatology, Hebei Medical University.

Patients were referred to the maxillofacial radiology department for CBCT imaging by their dentists to aid in diagnosis [19]. The inclusion criteria are patients who come to our hospital for oral implant surgery, orthodontic treatment for dental malformations, wisdom tooth extraction, and other treatments that require CBCT examination. The patients with mental illnesses and cognitive impairments had been excluded. All the volunteers were informed of the study, and had signed the consent form. These patients constituted the study population. In addition to assessing clinical symptoms, CBCT images (acquired using a KaVo 3D eXam, USA) were used to examine the structure, length, and medial angulations of the styloid process in order to investigate the incidence and characteristics of Eagle's syndrome (ES) in this patient population. The CBCT examinations were conducted with a 120 kV, 5 mA, 8.9 s exposure time and FOV:16 cm × 13 cm. Reference points on the patients' faces (center line, Frankfort horizontal plane, and condyle guide light) were used for positioning [20]. Only high-quality scans without any issues such as scattering or inaccuracies in bony borders were included in the study. The length and angle values were recorded separately for each side, as there were cases where differences existed between the right and left sides. All parameters related to the styloid process on one side of each patient were measured by the same radiologist, and each value was measured three times to obtain an average.

**Fig. 1:**
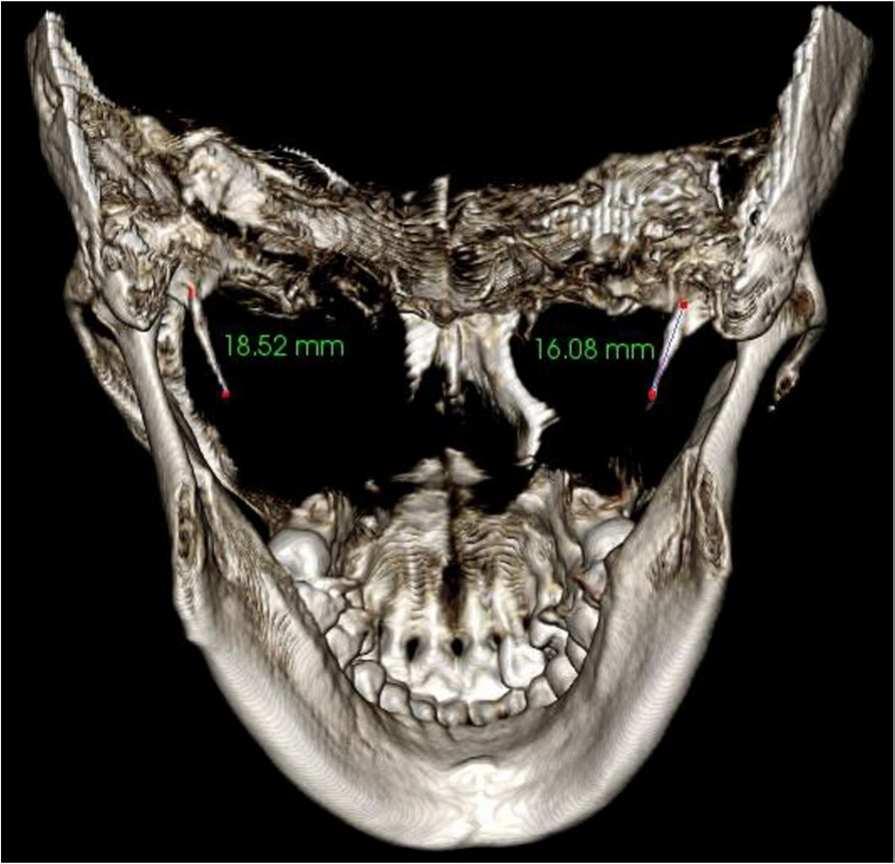
The measurement of the styloid process length

**Fig. 2:**
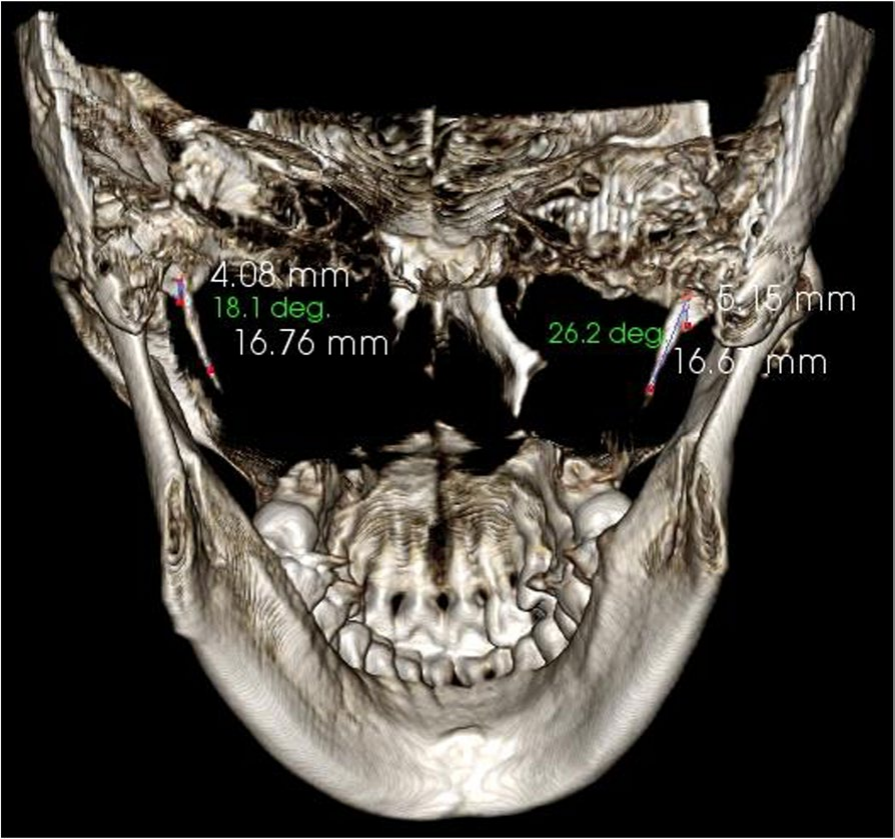
The measurement of the styloid process internal oblique angle and anterior oblique angle

**Fig. 3:**
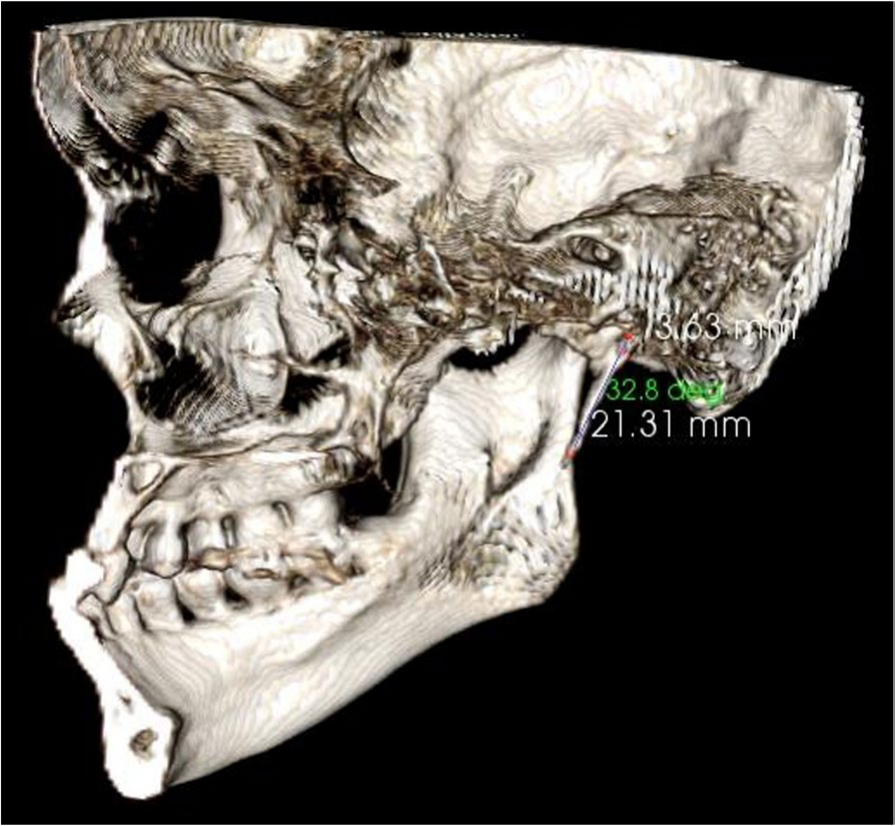
The measurement of the styloid process anterior oblique angle

**Fig. 4:**
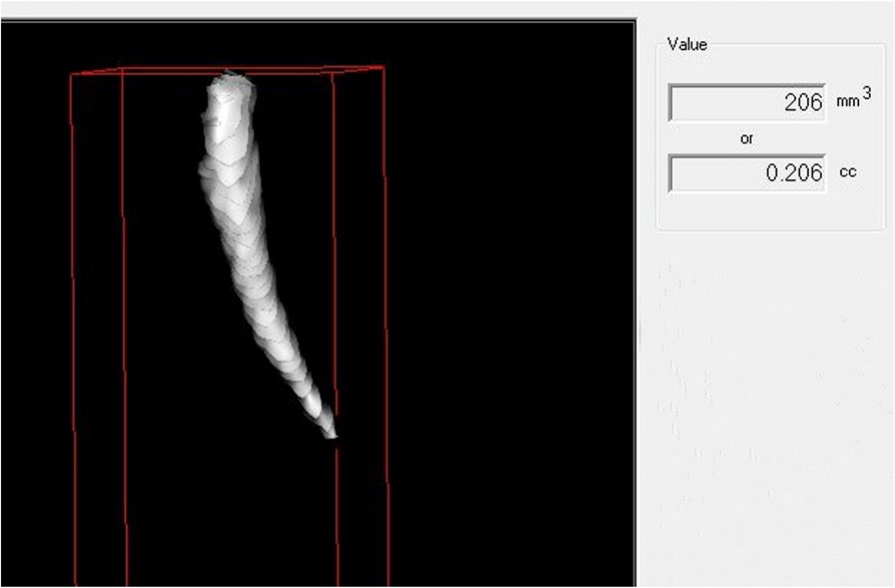
The measurement of the styloid process volume

The average lengths and angles of the styloid processes were evaluated. The length of the styloid process (Fig. 1) was measured from the caudal margin on the tympanic pleat to the tip of the process. If the cranial part of the styloid process was not visible, the length between the probable attachment point to the calvaria and the tip of the styloid process was measured. The angles (Figs. 2, 3) between the line connecting the base of both styloid processes and the volume (Fig. 4) of the styloid processes were also measured.

### Statistical analysis

Statistical analyses were performed using R version 3.6.3. The R package ggstatsplot (https://indrajeetpatil.github.io/ggstatsplot/index.html) was used for both statistical analyses and creating graphics. To assess the difference between the male and female groups, Welch's t-test was conducted. Correlations between different measurements were evaluated using Pearson's correlation coefficient. A significance level of 0.05 was used, meaning that a correlation was considered significant if the p-value was equal to or lower than 0.05.

## Results

This study was performed as a retrospective analysis on digital radiographs of 498 (241 males and 257 females) Chinese people between 20 and 40 years old. CBCT scan of styloid process suggested the mean length of right and left side were 29.21 mm (± 9.91 mm) and 28.67 mm (± 10.29 mm) respectively.

**Fig. 5:**
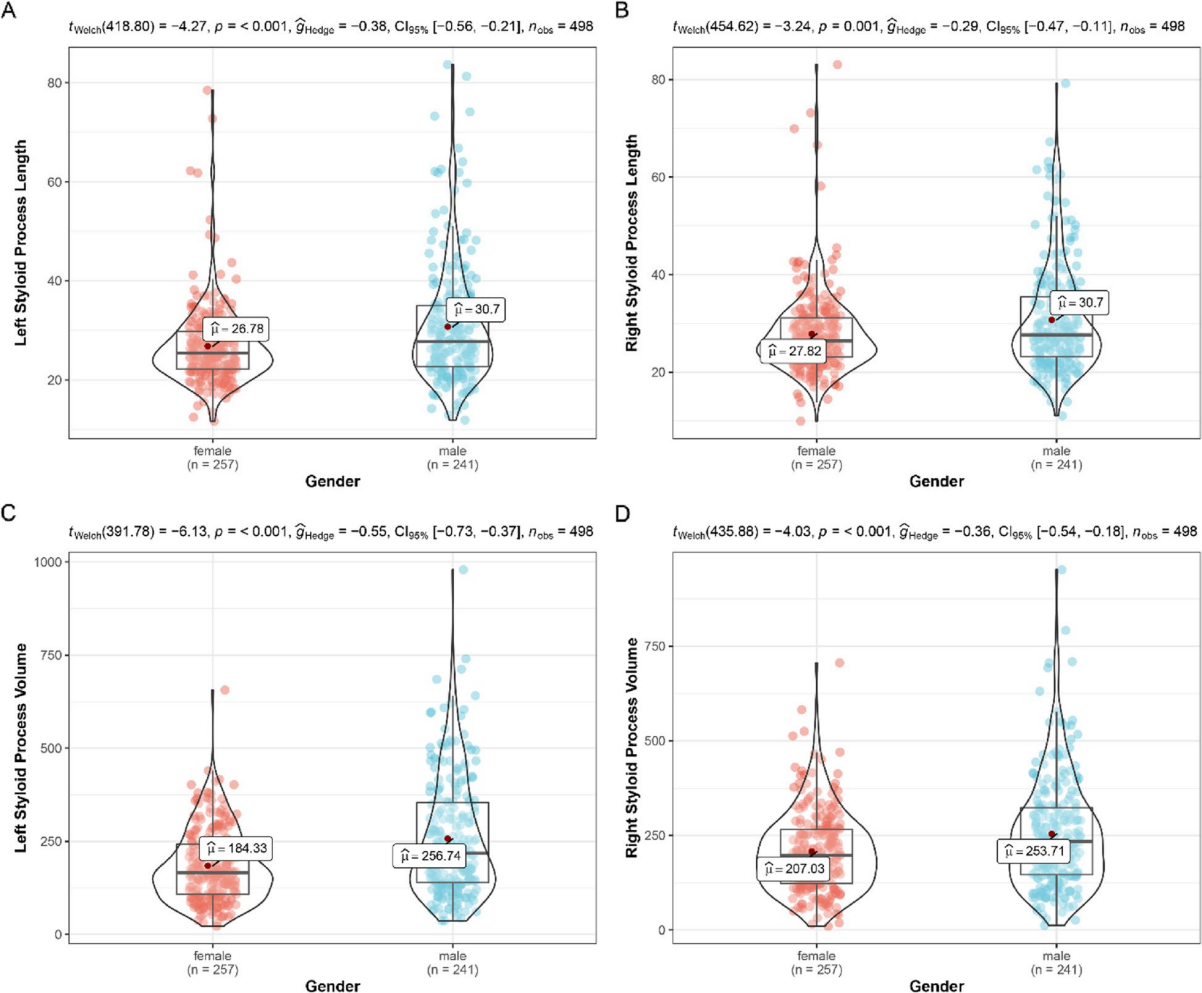
Styloid process length (**A**, **B**) and volume (**C**, **D**) distribution in males and females

Table 1 shows the subjects split into the gender groups and length, volume, internal oblique angle and anterior oblique angle of both sides had been measured. Compared to female group, male group displayed higher length of styloid process both in the left and right sides (Fig. 5). The mean length of left side in males was 30.7 mm (± 11.91 mm), whereas that in females was only 26.78 mm (± 8.08 mm), which showed significant differences (*p* < 0.001). Similarly, the mean length of right side differed significantly between male 30.7 mm (± 10.97 mm) and female 27.82 mm (± 8.6 mm) subjects (*p* < 0.001). As shown in Fig. 1 C and D, the mean volume of both sides in males were significantly higher (*p* < 0.001) than those in female (left: 256.74 mm^3^ ± 157.85 mm^3^ vs. 184.33 mm^3^ ± 96.21 mm^3^; right: 253.71 mm^3^ ± 147.2 mm^3^ vs. 207.03 mm^3^ ± 106.81 mm^3^).

**Table 1:** .

	Female (*n* = 257)		Male (*n* = 241)	
	Mean	SD	Mean	SD
Left_Length (mm)	26.78	8.08	30.7	11.91
Right_Length (mm)	27.82	8.6	30.7	10.97
Left_Volume (mm^3^)	184.33	96.21	256.74	157.85
Right_Volume (mm^3^)	207.03	106.81	253.71	147.2
Left_Internal_Oblique_Angle (°)	21	5.91	18.94	6.36
Right_Internal_Oblique_Angle (°)	24.15	6.04	21	5.79
Left_Anterior_Oblique_Angle (°)	30.84	8.83	30.67	8.11
Right_Anterior_Oblique_Angle (°)	29.96	8.86	28.95	7.53

**Fig. 6:**
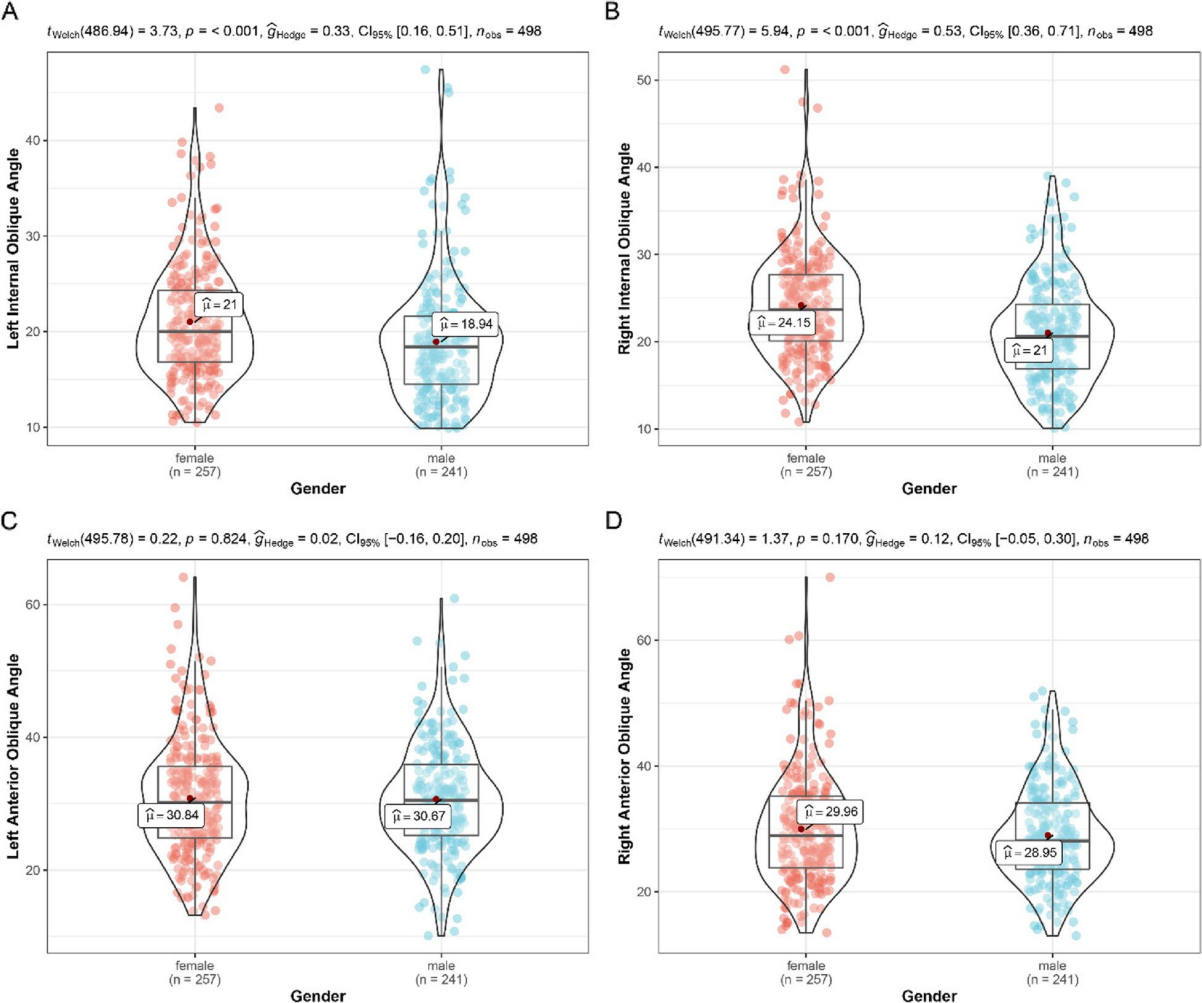
Styloid process angulations distribution in males and females

The mean internal oblique angle of left side differed between female 21° (± 5.91°) and male 18.94° (± 6.36°) subjects and displayed statistically significant (*p* < 0.001; Fig. 6). A similar pattern could be found in right side (female 24.15° ± 6.04° vs. male 21° ± 5.79°). Interestingly, the mean anterior oblique angle of left side was very close to 30.7°, both in male 30.67° (± 8.11°) and female 30.84° (± 8.83°) group. As regards right side, the mean anterior oblique angle of females 29.96° (± 8.86°) was slightly larger than that of males 28.95° (± 7.53°), but not statistically significant (*p* = 0. 17).

**Fig. 7:**
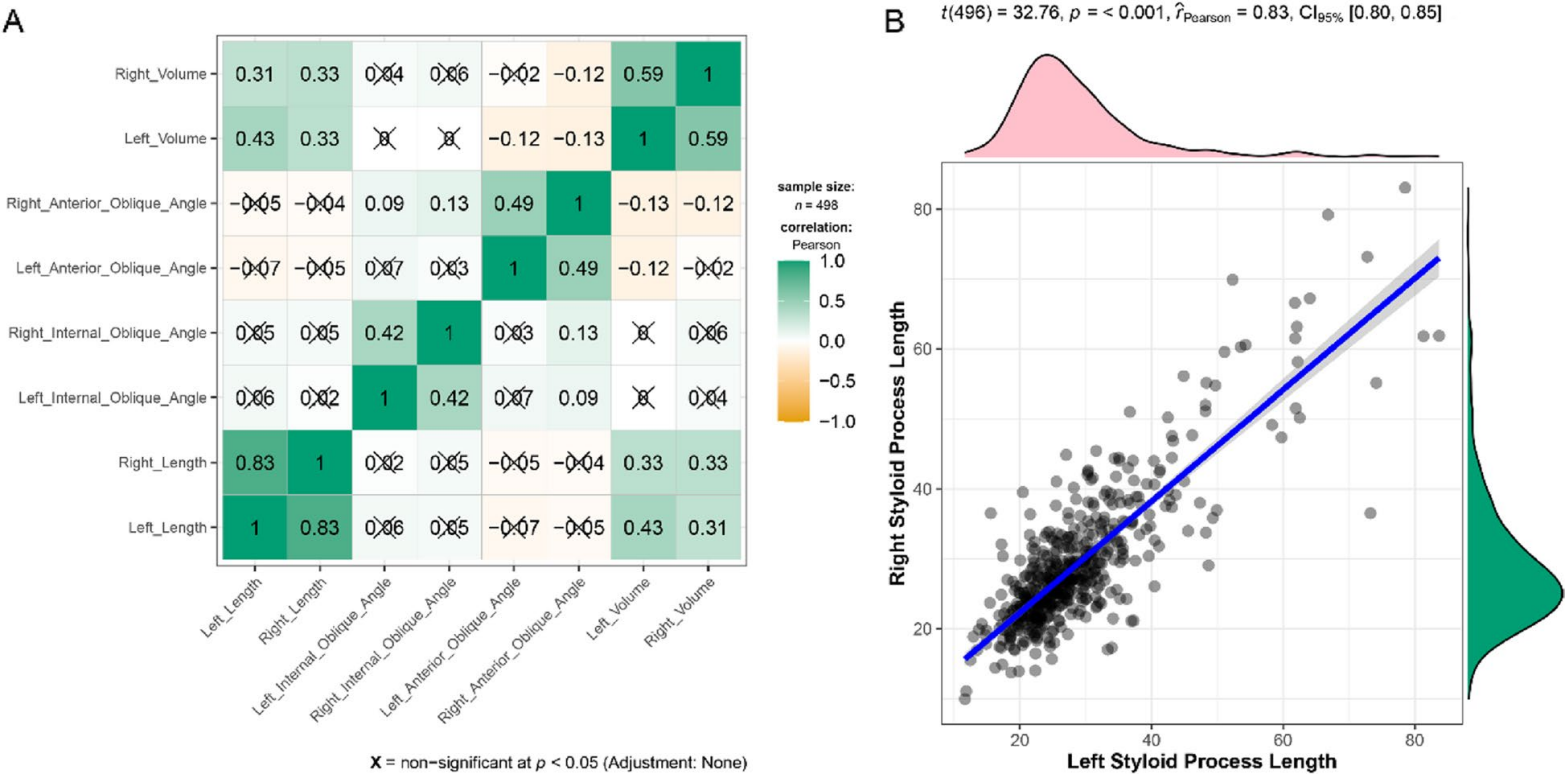
Correlations between different measurements

For evaluating the correlation between these different measurements, Pearson correlation test is used. As shown in Fig. 7, a strong positive linear relationship was found between left and right sides’process length (*r* = 0.83; *p* < 0.001). Not surprisingly, this positive relationship was also found between left and right sides’ process volume (*r* = 0.59; *p* < 0.05). In addition, length and volume of styloid process displayed a positive linear relationship, thought all the Pearson’s correlation coefficients were less than 0.5 (*p* < 0.05). Internal oblique angle of left side was in association with that of right side (*r* = 0.42; *p* < 0.05). Similarly, a weak positive linear relationship was observed between the left and right anterior oblique angle (*r* = 0.49; *p* < 0.05).

**Fig. 8:**
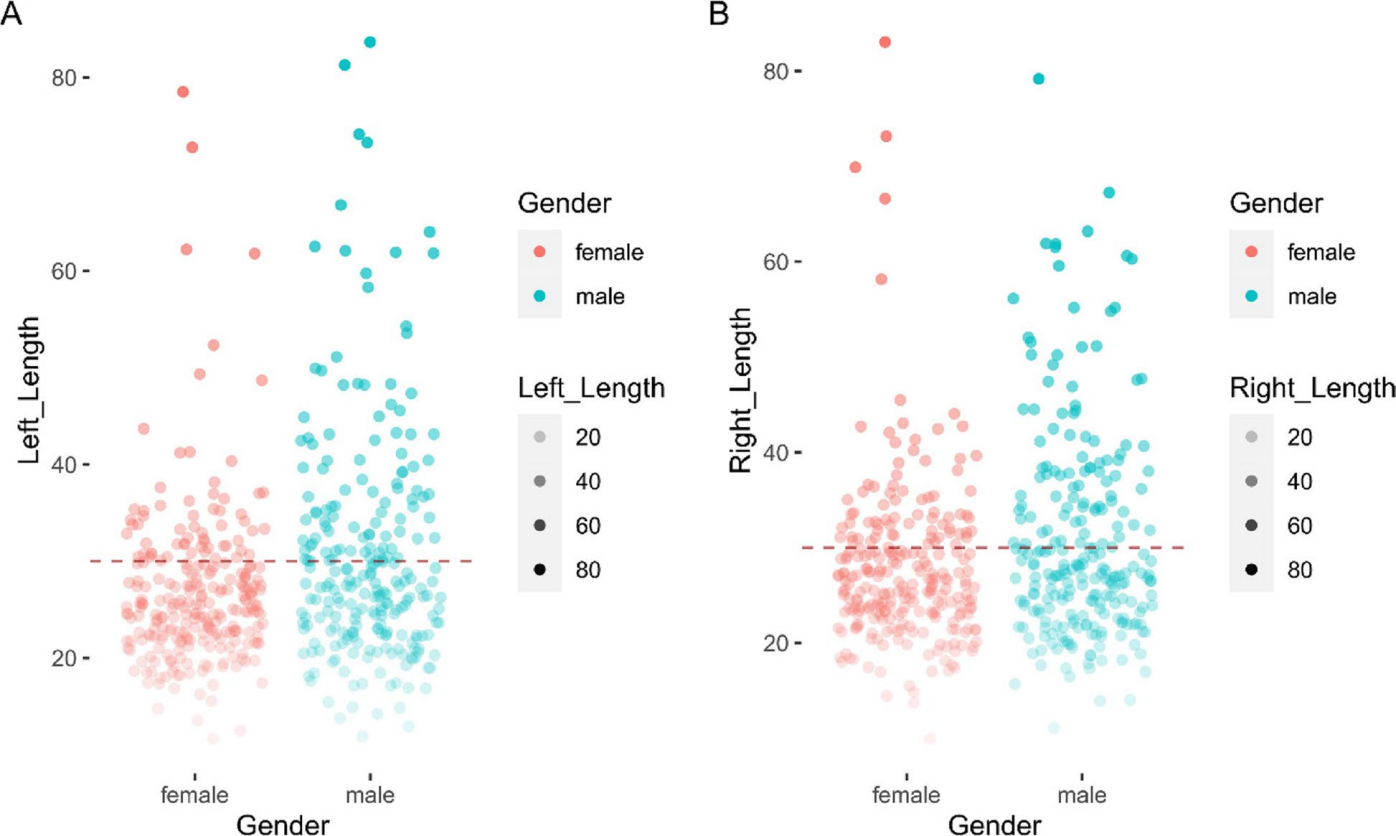
Elongated styloid process distribution in males and females with cutoff 30 mm (red dashed line)

Figure 8 and Table 2 provide information on the length of the styloid process, Elongated styloid process is defined as the condition at which the SP exceeds 30 mm when measured from the emergency point in the temporal bone down to the tip of the process considering a cutoff point of 30 mm [16, 21, 22]. It is shown that 62(24.13%) females and 101(41.91%) males had an elongated left styloid process (≥ 30 mm), while 75 (29.18%) females and 90 (37.34%) males had an elongated right styloid process. Overall, the prevalence of elongated styloid process was 32.73% in the left process and 33.13% in the right process.

**Table 2:** .

	Female	Male	All
Left process(> = 30 mm)	62(24.13%)	101(41.91%)	163(32.73%)
Right process(> = 30 mm)	75(29.18%)	90(37.34%)	165(33.13%)

## Discussion

The objective of the present study was to evaluate the incidence of radiological alterations in the length and sagittal angulation of the styloid process in individuals based on CBCT. Typically, panoramic radiographs are employed to ascertain whether the styloid process is elongated [16, 17]. Accurate assessment using 2D radiographic examinations is challenging due to considerations of projection geometry and the superimposition of the mandible and teeth on the styloid process. Regarding metric evaluation, panoramic techniques may induce distortions in the length and angulation of the styloid process [17]. This raises the inquiry of which medical imaging technique is most accurate for visualizing the styloid process. Clinical manifestations of Eagle Syndrome (ES) correlate with the dimensions of the stylohyoid complex [16]. Therefore, the complete length of the styloid process must be visualized for precise measurement. Although 3D imaging techniques, such as CBCT, involve higher radiation exposure, they yield the most comprehensive data concerning styloid length, angle, and degree of mineralization [8, 23]. Thus, CBCT is regarded as a valuable complement to 2D imaging techniques [24].

To explore the relationships between styloid process length, elongated styloid process (ESP), and ES, the distribution of styloid process lengths in males and females should be examined. The present study revealed a significant difference in the average length of the styloid process between male and female patients on both the left and right sides (Fig. 4; *p* < 0.001), indicating a strong correlation between gender and styloid process length, consistent with previous studies [8, 25, 26]. Similarly, the mean volume of both sides in males was significantly greater (*p* < 0.001) than in females. To assess the correlation between the mean lengths of the left and right sides, a strong positive linear relationship was identified (*r* = 0.83; *p* < 0.001), based on Pearson’s correlation coefficient. Furthermore, clinical symptoms of ES also depend on the angulation of the styloid process as well as its length [3]. These parameters can only be accurately measured using advanced imaging techniques. When the angle of the styloid process is narrow, it may be hypothesized to generate complaints due to compression of adjacent structures [27]. The present study indicated that males had significantly narrower internal oblique angle values than females (Fig. 5; *p* < 0.001), while no significant differences in anterior oblique angles were observed between genders. Research on the angulation of the styloid process is currently limited, necessitating further studies to establish the threshold of angulation that indicates an increased risk of developing ES.

Styloid processes are classified into two types based on size: normal and elongated. The threshold for elongation is highly variable, but many publications consider 30 mm as the standard cutoff [16, 21, 22]. The results of the present study demonstrated that the ratio of ESP in males was higher than in females (Fig. 8 and Table 2). This discrepancy arises primarily from employing the same cutoff (30 mm) for both genders to identify ESP, although males, on average, exhibited significantly longer styloid processes than females (Fig. 5 and Table 1). Additional data regarding the styloid process should be analyzed to determine a more reasonable threshold for elongation in females. Our study also indicated that approximately 33% of the study population had ESP, potentially contributing to patient complaints. However, the incidence of ESP remains contentious in the literature, with reported rates ranging from 1.4% to 30% [7, 16, 28]. The incidence of ES is considerably lower than that of ESP, with only a small percentage (between 1 and 5%) of patients reported as symptomatic [29, 30].

Therefore, further clinical studies, including patients with ESP but without pain complaints, are necessary to evaluate the precise correlation between styloid process length and neurological symptoms.

## Conclusions

This study presents the CBCT as an alternative method to CT or panoramic radiographs for the measurement and the assessment of the styloid process. Within the study in 498 subjects in China, it was observed that the males, on average, had significantly longer styloid process and narrower internal oblique angle than females either in left or right side. Moreover, around 33% of the study population had ESP. Further studies with three-dimensional radiographic techniques including greater numbers of people may be useful to find the reasonable thresholds in length and angulation of styloid process, which have strong correlation with clinical symptoms of ES, for different gender.

## Supplementary Information

Supplementary Material 1.

## Data Availability

Data is provided within the manuscript or supplementary information files.

## Abbreviations

SP: Styloid process
CBCT: Cone-beam computed tomography
ICA: Internal carotid artery
ECA: External carotid artery
TMD: Temporomandibular disorder
ESP: Elongated styloid process
ES: Eagle's syndrome
